# An edge communication based probabilistic caching for transient content distribution in vehicular networks

**DOI:** 10.1038/s41598-023-30315-6

**Published:** 2023-03-03

**Authors:** Divya Gupta, Shalli Rani, Basant Tiwari, Thippa Reddy Gadekallu

**Affiliations:** 1 Department of Computer Science and Engineering, Chandigarh University, Mohali, 140413 India; 2 Chitkara University Institute of Engineering and Technology, Chitkara University, Rajpura, Punjab 140401 India; 3 Hawassa University, Hawassa, Ethiopia; 4 School of Information technology and Engineering, Vellore Institute of Technology, Vellore, India; 5 Department of Electrical and Computer Engineering, Lebanese American University, Byblos, Lebanon

**Keywords:** Electrical and electronic engineering, Batteries

## Abstract

Vehicular Content Networks (VCNs) represent key empowering solution for content distribution in fully distributed manner for vehicular infotainment applications. In VCN, both on board unit (OBU) of each vehicle and road side units (RSUs) facilitate content caching to support timely content delivery for moving vehicles when requested. However, due to limited caching capacity available at both RSUs and OBUs, only selected content can be cached. Moreover, the contents being demanded in vehicular infotainment applications are transient in nature. The transient content caching in vehicular content networks with the use of edge communication for delay free services is fundamental issue and need to get addressed (Yang et al. in ICC 2022-IEEE international conference on communications. IEEE, pp 1–6, 2022). Therefore, this study focuses on edge communication in VCNs by firstly organizing a region based classification for vehicular network components including RSUs and OBUs. Secondly, a theoretical model is designed for each vehicle to decide its content fetching location (i.e. either RSU or OBU) in current region or neighboring region. Further, the caching of transient contents inside vehicular network components (such as RSU, OBU) is based on content caching probability. Finally, the proposed scheme is evaluated under different network condition in Icarus simulator for various performance parameters. The simulation results proved outstanding performance of the proposed approach over various state of art caching strategies.

## Introduction

Over the last several decades, rapid growth in the mobile network technology has led to remarkable explosion for mobile data. As predicted by cisco report, the mobile data traffic will reach 10.2 exabytes/month in 2025^1^. With the tremendous growth in mobile as well as electronics technology, soon the vehicular infotainment applications would also become progressively urging^2,3^. However, serving these highly distributed and dynamic content requests through a central cloud server is no longer suitable due to large traffic volume. The vehicular content networks (VCNs) address an encouraging and key empowering solution towards this objective by investigating vehicles and communicating devices (such as Road Side Units (RSUs) and Base Stations (BS)) to distribute contents in delay free manner and also by enabling vehicle-to-vehicle (V2V) communication^4–6^. Each vehicle in VCN is installed with On-Board-Unit (OBU) to have communication and caching facility. The RSU also has buffer storage to cache required contents and then can distribute it to the vehicle based on demand. The VCN leverages V2V communication facility among different OBUs to support content delivery in fully distributed manner^7^. However, caching all the contents on each vehicle is not feasible due to limited caching space available to each OBU and therefore VCN rely on edge based caching deployed at both RSUs and OBUs to offer Vehicular Edge Networks (VENs)^8–10^. Moreover, nowadays most of the content are transient in nature i.e. those have finite temporal validity (such as parking lot availability, traffic jam notifications, maps information etc.)^11,12^. Based on this discussion, in VEN, the timely delivery of requested content is hard to be guaranteed due to a) limited caching capacity of network nodes (either OBU or RSU), b) highly dynamic nature of road vehicles, c) transient content nature and d) short interval connections caused by limited service coverage^13^. Therefore, considering delay sensitive services in VENs, an enhanced caching and content delivery scheme is needed to fully explore the available network architecture with listed conditions. Unlike existing works on caching^14,15^, which mainly emphasize on getting content either from fixed RSUs or from nearby OBUs , this study along with the strategy of deciding content retrieval by requester from its current region or neighboring region to reduce the content retrieval delay also proposes a content caching scheme to store transient content based on content caching probability to reduce content redundancy and to better utilize available network resources.

Firstly, we perform region based classification on the network components based on the service coverage area of each region. Secondly, with the help of theoretical analysis on delay involved in fetching content, we model a strategy for a vehicle to decide from where it should retrieve the desired content to get minimum transmission latency. At last, we propose a content caching strategy to explore the transient content availability in neighbor nodes using content caching probability measure. In nutshell, the major contributions of this study are listed as following:

1. We organize a region based classification for vehicular network components including RSUs and moving vehicles (OBUs) based on service coverage area of each region. This classification not only improves content caching diversity inside specific region, but also enhances fast content delivery by offering reduced transmission delay.
2. We model a strategy for each vehicle to decide whether to fetch the requested content from its current region or from next neighboring region. Further, the fetching of content response in any region could be possible either through neighboring OBUs or from fixed RSUs present in current region based on the transmission delay involved in fetching requested content and connection handover.
3. We also propose a content caching strategy for caching of transient contents in VENs. This scheme is specifically designed to enhance diverse contents availability in particular region with certain lifetime validity. The scheme privileges high probability for caching of contents with longer lifetime and high content popularity with the condition of its low distribution in specific region.
4. We perform simulations in Icarus—an ICN caching simulator for evaluating the performance of the proposed caching strategy against various benchmark caching schemes used in literature. The result of simulations show improved performance of our proposed scheme for reduced content retrieval delay and high cache hit ratio along with minimized hop count.

The remaining of this paper is described as follows: “Related work” discusses the related works performed in the field of content caching for vehicular networks. “System model” describes the network model and content model used for the proposed scheme. “Request processing and content delivery” provides detail description on the request processing, content fetching and delivery procedure followed by the proposed scheme. The caching of transient contents inside network nodes based on caching probability is further described in “Content caching”. The evaluation related tasks such as scenario preparation, parameter selection and experimental simulation has been carried out in “Evaluation scenario”. In last, the conclusion of this study is presented in “Conclusion”.

## Related work

An efficient content caching scheme is significant for better network performance in vehicular environment. There have been number of studies focusing on VCNs and caching algorithms for providing meaningful results. The performance comparison of existing studies has been presented in Table 1. The work in^16^ with the aim of high user satisfaction and certain fairness value, proposed distributed heterogeneous service in P2P networks by considering content distribution, cache update and fairness in co-operation. However, the work did not take in to consideration the content location prediction measure. Some studies focus on content caching utilising an OBU (On Board Unit). The authors in^17^ work towards caching popular contents using cooperative caching scheme based on mobility prediction. To support content timely delivery in highly mobile vehicular environment, the mobility of vehicles is utilized to serve as relays. They propose predicting vehicle’s chance of accessing different hot spot regions using the Partial Matching technique (PPM). The proposed scheme preferred vehicles with longer sojourn time for caching popular contents. However, this scheme does not work well for environment with RSU and OBU embedded components. The hot zone would change in a dynamic environment, and it lacks a real-time updating mechanism; the selection of cache nodes can also be improved, such as taking the nodes’ workload into account. Considering mobility again as a prime factor, Zhang et al.^18^ proposed mobility aware vehicular cache strategy in CCN, where mobile vehicles are referred as cache carriers for speedy content delivery. The proposed scheme is based on designing optimization model for reducing the overall network energy consumption. However, this scheme lacks scalability and reliability.

With the use of D2D communication, the work in^19^ proposed content delivery in vehicular social networks by utilizing parked vehicles. For better utilization of resources, the on road parked vehicles form vehicular social communities with the moving vehicles to satisfy the requests for contents located inside them. For V2V framework, Zhao et al.^20^ proposed community similarity and population based cache strategy to reduce cache redundancy, where a dynamic probability cache strategy is designed based on community similarity and selection of caching vehicle is made through hop count based on content popularity.

The work in^21^ proposed cluster based strategy for grouping vehicles with similar mobility patterns using predictions. Upon creation of clusters, co-operative caching is introduced to support inter-cluster and intra-cluster caching inside vehicles clusters. Further, content popularity is examined to perform caching. Van et al.^22^ proposed caching scheme in CCN for vehicular adhoc network applications based on network trait which include content popularity, link stability of vehicle, cache capacity and user preferences. Based on these parameters, each vehicle independently make content cache decisions.

**Table 1:** Comparison of proposed work with related work.

Ref	Strong point	Weak point	Comparison with our approach
^16^	Co-operative caching strategy, fairness sharing among vehicles	low scalability, No implicit estimation of user-satisfaction	Our proposed design is highly scalable and accurate
^17^	Partial matching method, considering sojourn time	Poor scalability and dynamic adaptability	We leverage the various dynamic factor to guarantee dynamic adaptability
^18^	Mobility aware architecture, reduced energy consumption	less considered content and network factors	We considred vehicle speed and content lifespan
^19^	utilizing cache resources of nearby vehicles	High overhead	We considered moving vehicles on road
^21^	Mobility prediction based group caching	NA	Our design considered content lifespan and distribution along with content popularity for caching
^22^	Focusing on content attributes	Lacking the influence of vehicles’ motion	We have a comprehensive utilization of network factors

Even though in past times, a lot of work has been done on content distribution in vehicular environment, still, efficient and cooperative caching inside OBU and RSU needs further studies. In particular, existing works focused on impact of number of requests from vehicles and contents being cached in their own OBU’s on caching strategy. However, the study on dynamic caching scheme by jointly considering vehicle’s requests, mobility, location and content lifetime is need of the hour.

## System model

### Terminology

The notations and terminology used in this paper for content caching and fetching strategy is presented in Table 2. To ensure the availability and reliability of the varying parameters due to mobility, the entire network time period is divided in to several time chunks, where each chunk is of T seconds. The information gathered from the previous chunk $T_{past}$ can be used for current chunk $T_{current}$ based on requirement.

### Network model

**Figure 1:**
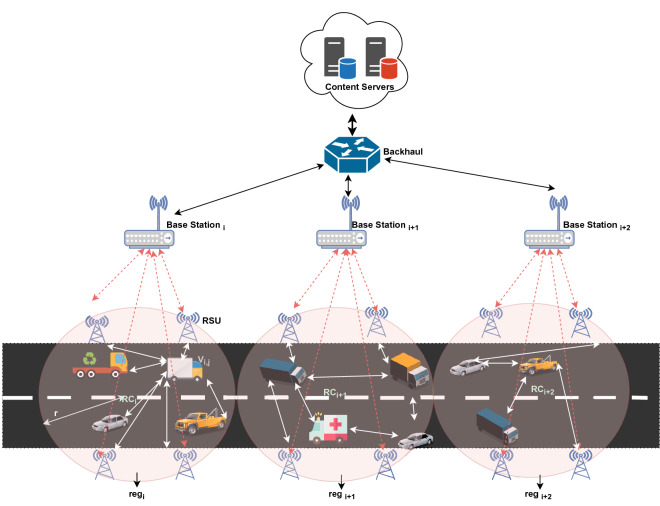
Region oriented network architecture.

The vehicular network is mainly comprises of different moving vehicles and various RSUs deployed on the road. We organize region based classification on the road where a region covers several connected RSUs through BS and RSUs in different regions are not connected (refer Fig. 1). The services of any specific region say $reg_i$ are limited with in coverage area of radius $r_i$ and center point $RC_i$. The vehicles in each region are equipped with wireless communication and storage device i.e. OBU. In case of any request for a content by moving vehicle, if replica of the content is placed in its own OBU, then replica is provided locally satisfying $\theta _{local-hit}=1$. Otherwise, request has to be satisfied by either fixed RSUs or neighbouring OBUs present in requested vehicle’s current region or by the network components in neighbour region on the basis of delay involved in fetching the content. The BS act as a intermediate node between several RSUs and central core network. Each BS maintains the information of its connected region in form of different tables which includes Sojourn Time Table (STT), Location Table (LT) and Vehicle Speed Table (VST) (refer to Tables 3, 4, 5). The central core network is a remote server with huge caching capacity and processing speed which stores all content items in its cache queue. The communication between core network and BS is only possible through backhaul network.

**Table 2:** Notations and terminology.

Symbol	Definition
$reg_i$	The specific region *i*
$r_i$	Coverage area of $reg_i$
$RC_i$	Center point of $reg_i$
*S*	The number of regions
$V_{i,j}$	The vehicle *j* in $reg_i$
$R_{i,m}$	The RSU *m* in $reg_i$
$Speed_{i,j}$	The speed of vehicle $V_{i,j}$
$\lambda _{i,j}$	The request rate of $V_{i,j}$
$c_k$	The request for any content *k*
*M*	The total number of available contents
$P_{c_k}$	The probability of content $c_k$
*C*	The set of available contents
$TR_{i,j}$	The transmission range of $V_{i,j}$
$V_{i,p}$	The vehicle in transmission range of $V_{i,j}$
$Prob_{i,p}$	The probability of request generated by $V_{i,p}$
$treq_i$	The total number of requests from all the moving
	Vehicles with in coverage area of $reg_{i}$
$Cand_{p,c_k}$	The potential candidate *p* to serve $c_k$ to $V_{i,j}$
$CP_{p,c_k}$	The provider *p* for content $c_k$ to $V_{i,j}$
$CT_{V_{i,j},V_{i,p}}$	The possible connection time between $V_{i,j}$ and $V_{i,p}$
$Del_{V_{i,j}, V_{i,p}}$	The content retrieval delay between $V_{i,j}$ and $V_{i,p}$
$AD_{c_k}$	The average delay to fetch content $c_k$ from other
	Vehicles in transmission range of $V_{i,j}$
$Del_{R_{i,m},V_{i,j} }$	The delay involved in getting $c_k$ from any one of
	The RSU say $R_{i,m}$ to $V_{i,j}$
$HC_{R_{i,m},V_{i,j}}$	The number of hops between $R_{i,m}$ to $V_{i,j}$
$BW_{R_{i,m},V_{i,j}}$	The available bandwidth between $R_{i,m}$ and $V_{i,j}$
$MD_{i,j}$	The moving distance for vehicle *j* in region *i*
$(x_{i,j}^{in}, y_{i,j}^{in})$	Entering co-ordinates of vehicle *j* in $reg_i$
$(x_{i,j}^{out}, y_{i,j}^{out})$	Leaving co-ordinates of vehicle *j* from $reg_i$
$T_{s({i,j}})$	Sojourn time of vehicle *j* in region *i*
$LS(c_k)$	The lifespan of content $c_k$
$FP(c_k)$	The freshness period of content $c_k$
$OP(c_k)$	The originating period of content $c_k$
$\theta _{LS}$	The current value of lifespan threshold
$\theta _{LS,old}$	The previously calculated threshold value
$Pop(c_k)$	The popularity of the content $(c_k)$
$CCP(c_k)$	The caching probability for content $(c_k)$

**Table 3:** Range of the sojourn time.

**SST**				
VID	$V_{i,1}$	$V_{i,2}$	$V_{i,j}$	.
Sojourn Time	$T_s(i,1)$	$T_s(i,2)$	$T_s(i,j)$	.

**Table 4:** Range for in-out coordinates.

**LT**				
VID	$V_{i,1}$	$V_{i,2}$	$V_{i,j}$	.
IN co-ordinates	$(x_{i,1}^{in}, y_{i,1}^{in})$	$(x_{i,2}^{in}, y_{i,2}^{in})$	$(x_{i,j}^{in}, y_{i,j}^{in})$	.
OUT co-ordinates	$(x_{i,1}^{out}, y_{i,1}^{out})$	$(x_{i,2}^{out}, y_{i,2}^{out})$	$(x_{i,j}^{out}, y_{i,j}^{out})$	.

**Table 5:** Average speed of the vehicles.

**VST**				
VID	$V_{i,1}$	$V_{i,2}$	$V_{i,j}$	.
Average Speed *km*/*h*	$speed_{i,1}$	$speed_{i,2}$	$speed_{i,j}$	.

The RSUs are located on different places on the edge of the network and different kind of vehicles are moving on the road. Within the coverage area of region i ($reg_i$) where $i \in S = \{1, \ldots , S\}$, there could be multiple moving vehicles where $V_{i,j}$ represents *jth* vehicle in $reg_i$. The speed of vehicle $V_{i,j}$ is denoted by $speed_{i,j}$. The speed of different vehicles will fall in to range $[min(speed_{i,j}), max(speed_{i,j})]$.

### Content model

Without the loss of generality, we consider the content arrival requests always follows poisson distribution. The contents are requested by moving vehicles with request rate $\lambda _{i,j}$. Each vehicle can request any content $c_k$ from total number of available contents M $(1 \le k \le M)$. The core network stores original content and the replicas of content may be cached in different RSUs and OBUs based on defined caching strategy. Due to limited cache capacity available with RSUs and OBUs, it can perform only partial caching. The contents in the network undergoes different popularity index based on the fact that some contents are requested more frequently than others. Based on mathematics, the probability of content $c_k$ to be requested by moving vehicle $v_{i,j}$ is computed as^23^:

1 $$\begin{aligned} P_{c_k} = \frac{1}{\left[ \sum _{k=1}^M \frac{1}{e^{\frac{\wp }{c_k}}}\right] *{e^{\frac{\wp }{c_k}}}} \end{aligned}$$

where $\wp$ is the zipfian distribution parameter and $e^{c_k}$ is the ranking of number of requests for $c_k$.

## Request processing and content delivery

The request for any content say $c_k$ ($c_k \in C =\{ c_1, c_2, \ldots , c_M\}$) by vehicle $V_{i,j}$ can be served locally if $OBU_{i,j}$ caches content replica, otherwise there are two options for request processing (1) to fetch content from neighbouring vehicles in current region which caches content $c_k$ or to contact the RSUs in current region which caches $c_k$. (2) To contact the neighbouring region $reg_{i+1}$ to get the required content $c_k$.

### Delay to obtain content from neighbouring OBUs

The maximum possible vehicles to which a vehicle $V_{i,j}$ can contact is determined by its fixed transmission range $TR_{i,j}$. Considering $V_{i,p}$ as one of the vehicle among all the vehicles which $V_{i,j}$ is able to connect within $TR_{i,j}$, the number of requests from $V_{i,p}$ in past time frame $T_{past}$ can be calculated as

2 $$\begin{aligned} req_{i,p} =\sum _i Prob_{i,p} *treq_i \end{aligned}$$

where $treq_i$ is the total number of requests from all the moving vehicles with in coverage area of $reg_{i}$ in past time frame $t_{past}$ , $Prob_{i,p}$ is the probability of request generated by $V_{i,p}$ with in coverage of $reg_{i}$ and is calculated as

3 $$\begin{aligned} Prob_{i,p}= \frac{\lambda _{i,p}}{\sum _{i} \sum _{p}\lambda _{i,p}} \end{aligned}$$

Here, $\lambda _{i,p}$ is request rate of vehicle $V_{i,p}$

Using Eq. (2), if the request for content $c_k$ would be made by $V_{i,p}$ in $t_{past}$ and caching probability of $c_k$ is large enough as discussed in next section, then $c_k$ might be present inside $OBU_{i,p}$ as vehicle caches replica of content after fetching it. Based on this, $V_{i,p}$ will be a potential candidate to serve $c_k$ to $V_{i,j}$ if

4 $$\begin{aligned} Cand_{p,c_k}= {\left\{ \begin{array}{ll}1, \quad \sum _i Prob_{i,p} *treq_i * P_{c_k} \ge 1 \\ \\ 0, \quad \sum _i Prob_{i,p} *treq_i * P_{c_k} < 1 \end{array}\right. } \end{aligned}$$

Here, $Cand_{p,c_k}$=1 means, the content $c_k$ is cached in the $OBU_{i,p}$ of vehicle $V_{i,p}$.

In case of delay offered to fetch content $c_k$ from $V_{i,p}$ is less than the possible connection time $CT_{V_{i,j},V_{i,p}}$ between $V_{i,j}$ and $V_{i,p}$, then $OBU_{i,p}$ will become a provider for content $c_k$ to $V_{i,j}$. Therefore, we have

5 $$\begin{aligned} CP_{p,c_k} = {\left\{ \begin{array}{ll} 1, \quad Del_{V_{i,j}, V_{i,p}} \le CT_{V_{i,j},V_{i,p}} \\ \\ 0, \quad Del_{V_{i,j}, V_{i,p}} > CT_{V_{i,j},V_{i,p}} \end{array}\right. } \end{aligned}$$

Here, $Del_{V_{i,j}, V_{i,p}}$ represents delay offered in getting requested content by $V_{i,j}$ from $V_{i,p}$. Using Eqs. (4) and (5), we can calculate average delay $AD_{c_k}$ to fetch content $c_k$ from other vehicles in transmission range of $V_{i,j}$ as

6 $$\begin{aligned}{} & {} D_{c_k} = \sum _{p=1}^ W CP_{p,c_k} * Cand_{p,c_k} *Del_{V_{i,j}, V_{i,p}} \end{aligned}$$

7 $$\begin{aligned}{} & {} AD_{c_k} = \frac{D_{c_k}}{W-W'} \end{aligned}$$

where, W is the total number of vehicles in transmission range of $V_{i,j}$ in $reg_i$ and W$'$ is the count of vehicles among all W which do not satisfy either $CP_{p,c_k}$ =1 or $Cand_{p,c_k}$ =1.

### Delay to obtain content from region’s fixed RSUs

To obtain the requested content $c_k$ from the fixed RSU present in the $reg_i$ of moving vehicle $V_{i,j}$ which has cached replica of $c_k$, the delay involved in getting $c_k$ from any one of the RSU say $R_{i,m}$ to $V_{i,j}$ can be calculated as

8 $$\begin{aligned} Del_{R_{i,m},V_{i,j} } = \frac{Size(c_k)* HC_{R_{i,m},V_{i,j}}}{BW_{R_{i,m},V_{i,j}}} \end{aligned}$$

where $HC_{R_{i,m},V_{i,j}}$ is the number of hops between $R_{i,m}$ to $V_{i,j}$ in shortest path and $BW_{R_{i,m},V_{i,j}}$ defines the available bandwidth between $R_{i,m}$ and $V_{i,j}$. For simplicity, we assume all the network contents to have same size, i.e $size(c_k)$.

In case of delay offered to fetch content $c_k$ from $R_{i,m}$ is less than the possible connection time $CT_{R_{i,m},V_{i,j}}$ between $V_{i,j}$ and $R_{i,m}$, and $R_{i,m}$ caches replica of content $c_K$ provided

9 $$\begin{aligned} Cand_{R_{i,m},c_k}= {\left\{ \begin{array}{ll}1, \quad treq_{R_{i,m}} * P_{c_k} \ge 1 \\ \\ 0, \quad treq_{R_{i,m}} * P_{c_k} < 1 \end{array}\right. } \end{aligned}$$

Here, $Cand_{R_{i,m},c_k}$=1 means, the content $c_k$ is cached in the RSU $R_{i,m}$. $treq_{R_{i,m}}$ denotes total number of requests made with in transmission range of RSU *m* in region *i*.

The $R_{i,m}$ will become a provider for content $c_k$ to $V_{i,j}$. Therefore, we have

10 $$\begin{aligned} CP_{R_{i,m},c_k} = {\left\{ \begin{array}{ll} 1, \quad Del_{R_{i,m},V_{i,j} } \le CT_{R_{i,m},V_{i,j}} \\ \\ 0, \quad Del_{R_{i,m},V_{i,j} } > CT_{R_{i,m},V_{i,j}} \end{array}\right. } \end{aligned}$$

Using Eqs. (9) and (10), we can calculate average delay $AD_{c_k}$ to fetch content $c_k$ from other RSUs in transmission range of $V_{i,j}$ in $reg_i$ as

11 $$\begin{aligned} \frac{ \sum _{m=1}^ I CP_{R_{i,m},c_k} * Cand_{R_{i,m},c_k} *Del_{ R_{i,m},V_{i,j}}}{I-I'} \end{aligned}$$

where I is the total number of RSUs in transmission range of $V_{i,j}$ in $reg_i$ and I’ is the count of RSUs among all I which do not satisfy either $CP_{R_{i,m},c_k}$ =1 or $Cand_{R_{i,m},c_k}$ =1.

#### Decision on content fetching in current region

To retrieve content $c_k$ by $V_{i,j}$ in $reg_i$, if Eq. (12) holds true then content will be provided by neighbouring OBUs otherwise RSU will become content provider.

12 $$\begin{aligned} \frac{ \sum _{p=1}^ W CP_{p,c_k} * Cand_{p,c_k} *Del_{V_{i,j}, V_{i,p}}*HC_q}{W-W'} < min \left( \frac{ \sum _{m=1}^ I CP_{R_{i,m},c_k} * Cand_{R_{i,m},c_k} *Del_{ R_{i,m},V_{i,j}}}{I-I'}\right) \end{aligned}$$

Here, $HC_q$ denotes hop count to obtain $c_k$ from OBUs with multiple hops.

### Decision on content fetching from neighbouring region

For the content request $c_k$ by $V_{i,j}$, if the delay involved in getting $c_k$ from either neighbouring OBU or from fixed RSU in $reg_i$ is greater than the Connection Time (CT) i.e. $Del_{R_{i,m},V_{i,j} } > CT_{R_{i,m},V_{i,j}}$ and $Del_{V_{i,j}, V_{i,p}} > CT_{V_{i,j},V_{i,p}}$ for all possible m and p values, then request for $c_k$ cannot be granted by $reg_i$.The fact behind this is the moving speed of vehicle $V_{i,j}$. Considering request for $c_k$ by $V_{i,j}$ at time frame $t_0$ with moving speed $speed_{i,j}$, if the request has not been served with in sojourn time $T_{s({i,j}})$ (Stay time of vehicle *j* in region *i*), then $V_{i,j}$ will get changed to $V_{i+1,l}$ leaving $reg_i$ and entering $reg_{i+1}$. The $speed_{i,j}$ can be calculated using

13 $$\begin{aligned} speed_{i,j} = \frac{MD_{i,j}}{T_{s({i,j}})} \end{aligned}$$

Here, $MD_{i,j}$ is moving distance for vehicle *j* in region *i* and can be computed using values stored in Table 4. The value $(x_{i,j}^{in}, y_{i,j}^{in})$ represents entering co-ordinates and $(x_{i,j}^{out}, y_{i,j}^{out})$ represents leaving co-ordinates of $V_{i,j}$ for $reg_i$.

14 $$\begin{aligned} MD_{i,j} = \sqrt{(x_{i,j}^{in}- x_{i,j}^{out})^2 + (y_{i,j}^{in}- y_{i,j}^{out})^2 } \end{aligned}$$

When vehicle enters a new region $reg_{i+1}$, getting the content from previous region with high delay degrades network performance. Therefore, the request for content would be served in this new region $reg_{i+1}$ either by neighbouring OBUs or by fixed RSUs as discussed earlier. If delay is still greater than the connection time between $reg_{i+1}$’s RSUs and neighbouring OBUs, then request would be served by next $reg_{i+2}$ and so on.

## Content caching

The content caching in our proposed scheme mostly follows the conventional caching as in legacy NDN^24,25^. Each NDN router maintains three data structures: (a) Content Store (CS), (b) Pending Interest Table (PIT) and, (c) Forwarding Information Base (FIB). The CS is responsible for caching the content at router and act as storage buffer. PIT holds the entry for requests whose response has yet not been recorded. FIB is used to route the requests to next routers in forward direction. In NDN, upon receiving a data packet either by receiver or any intermediate node, it first queries in PIT . The decision on whether to cache the content or not gets initiated only if the matching name entry resides in PIT. As discussed in “Introduction” section, the most content requests are transient in nature and expires after certain period of time as well as have different popularity rates. Therefore, decision on caching the transient content is based on content caching probability which considers three caching parameters: (1) content lifespan, (2) content popularity and (3) content distribution. The content caching probability is computed at each RSU or moving vehicle based on the information stored in received data packet and corresponding interest packet.

The calculation of these caching parameters for computation of caching probability is described in following.

### Content lifespan

The transient nature of any content is reflected only through its lifespan value. Each data packet generated by the original content source carries some freshness value which denote the time up to which this content will remain valid. Using this, any potential cacher (either RSU or OBU) will mark content as resilient or non-resilient.

On receiving a data packet for content say $c_k$ by any vehicle $V_{i,j}$ or RSU $R_{i,m}$ in $reg_i$, its LifeSpan (*LS*) is computed as:

15 $$\begin{aligned} LS(c_k)= min \left( 1, \frac{RL_{c_k}}{\theta _{LS}} \right) \end{aligned}$$

where *RL* denotes the residual life of content $c_k$. The value of *RL* for any $c_k$ can be easily computed from the values present in data packet carrying $c_k$,

16 $$\begin{aligned} RL(c_k)= OP(c_k) + FP(c_k)- T_{current} \end{aligned}$$

where $OP(c_k)$ is originating period of content $c_k$, $FP(c_k)$ represents content’s freshness period and $T_{current}$ is current time.

The $\theta _{LS}$ represents the current value of lifespan threshold and get updated whenever any vehicle or RSU receives data packet. This computation is mainly based on the average residual of previously received data packets.

17 $$\begin{aligned} \theta _{LS} = (1- \mu )\theta _{LS,old} + \mu . RL(c_y) \end{aligned}$$

Here, $\mu \in (0,1)$ and is set to 0.125 to ignore high fluctuation in estimation and is set based on historic values. $\theta _{LS,old}$ represents previously calculated threshold value and $RL(c_y)$ provides residual lifespan of previously received content $c_y$ by this potential cacher.

From Eq. (15), the lifespan of any content may have any values between (0,1) inclusive. Only the contents with residual value higher than the current lifespan threshold will correspond to $LS = 1$ and therefore would be treated as resilient. Otherwise contents with $LS < 1$ is known as non-resilient.

### Content popularity

The popularity of the content $Pop(c_k)$ can be easily computed at any node by determining the number of requests received for content $c_k$ over the maximum number of request received for any possible content which belongs to set *C*.

18 $$\begin{aligned} Pop(c_k)= \frac{req(c_k)}{max(req(c_i))} \forall c_i \in C \end{aligned}$$

Using Eq. (18), each node maintain contents according to their popularity. The maximum value 1 of $Pop(c_k)$ denotes $c_k$ as the most requested content. For all other contents having less number of requests, the $Pop(c_k)$ will have values in range (0, 1).

### Content distribution

This parameter is used to measure the distribution of the content in the neighbourhood of the requested OBU. The high distribution value for any content $c_k$ indicates presence of its replicas in nearby nodes (either vehicles or RSUs). The value of this parameter is computed using hop count.

In particular, any node on receiving a data packet first looks for the hop count field present in the packet and calculates the content distribution $CD_(c_k)$ value as:

19 $$\begin{aligned} CD(c_k) = \frac{1}{HC(c_k)} \end{aligned}$$

From Eq. (19), the higher value of hop count $HC(c_k)$ for content $c_k$ signifies less distribution of content in the neighbourhood and vice versa. One important point to discuss here is that content distribution is not used to manipulate any network topology or any node’s state information. However, this parameter is used in our scheme just to calculate close availability of a content to any vehicle or RSU.

### Content caching probability

Any node in the network upon receiving a data packet for content $c_k$ decides on whether to cache the content or not based on content caching probability $CCP(c_k)$ and is computed using Eq. (20).

20 $$\begin{aligned} CCP(c_k)= [ 1- [1- Pop(c_k)] *CD(c_k) ]* LS(c_k) \end{aligned}$$

From Eq. (20), it is evident that caching probability is directly proportional to content lifespan. For the resilient contents i.e. when $LS(c_k) =1$, the caching probability only depends upon content popularity and its distribution.

Specifically, caching probability is higher for the contents which are most popular and are less distributed in the neighbourhood (i.e. $Pop(c_k) =1$ and $CD(c_k)=0$) and vice versa.

The caching probability is maximum for the contents with $LS(c_k) =1$, $Pop(c_k) =1$. As a whole, caching based on caching probability formula used in Eq. (20), ensures caching such contents at vehicle or RSU which are less available in the neighborhood. The advantage of using this scheme is to enhance content diversity and reduce content redundancy in specific region. The distinct contents get cached by distinct network nodes (either vehicles or RSUs) in a region which improves the chances of getting request response from nearby nodes with reduced response delay and less likely to approach original content source. Any node caching the content, caches it with content validity which is set equal to the content residual lifetime. As soon as the validity expires, the content gets removed from the CS of that node leaving space for new content to get in. For a new content to be cached in case of CS full, the content’s residual time and LRU are used in combination to perform eviction as it is highly suitable for transient content caching in vehicular environment.

## Evaluation scenario

By using simulations in the Icarus simulator^26^, the effectiveness of the suggested technique has been assessed. For research on ICN caching and routing, a simulator with four basic blocks-scenario generation, scenario orchestration, experiment execution, and result collection-was created. The network is initially warmed up with $3\times 10^5$ worth of queries before evaluation. Before evaluating system performance, network caching nodes are first provided warm up requests, which instruct them to cache content. After the network has finished its warm-up phase, measured requests with a $6\times 10^5$ value are issued to gauge network performance. Since the poisson distribution is the most popular distribution employed by different caching algorithms throughout implementation, each user sends request messages that adhere to it. The default setting for the network request rate in Icarus settings is 12 requests per second for the entire network. The popularity of the content follows the Zipfian distribution, with an alpha parameter that spans from 0.6 to 1.2. The value *alpha* in the Zipfian distribution represents the concentration of user choice. Large *alpha* values indicate stronger user preference concentration; for example, $alpha=1.2$ indicates that more users are interested in the same request than $alpha=0.6$^27^. Since GARR network architecture has been extensively used in recent publications for performance evaluation, it is used in the tests. The total caching capacity across all nodes is maintained to ensure storage capacity fairness. As a result, the total network caching capacity is divided by the number of contents to determine the uniform storage capacity for the aforementioned architecture. This study covers several scenarios by adjusting the network cache from.06 to 5% for testing purposes. Further to preserve uniformity, this technique considers all network nodes with homogeneous caching capacity i.e. each node with similar cache reserve. The replacing of content is equally as crucial as creating content placement scenarios. The proposed technique considers LRU for cache replacement due to its minimal complexity and good consistency with already known ubiquitous caching schemes. The studies have been run based on all the aforementioned conditions, taking into account 4 network cache capacities and 6 different content popularity (*alpha*) values. The simulations were run four times, with the average value for each performance indicator taken into account when contrasting the suggested strategy with other benchmark plans.The results were obtained at a 95% confidence level.

###  Performance metrics

This paper assesses the effectiveness of the suggested technique using the following metrics, which are among the many available for calculating the relevance of caching strategies^28,29^:

#### Cache hit ratio (CHR)

CHR is a measurement of the ratio of the number of interest packets that a cache can handle to the number of interests that are transmitted around the network. It essentially provides an estimate of router’s efficiency and may be determined using the formula given in below equation.

21 $$\begin{aligned} Cache Hit Ratio (CHR)= \frac{Cache_{hits}}{Cache_{hits}+ Cache_{miss}} \end{aligned}$$

#### Content retrieval delay (CRD)

It calculates in milliseconds and this metric measures the time taken to relates more explicitly to the intervals of time that a user must wait before receiving a response to a content request. Individual factors like excessive network congestion and a lack of diversity in the material can have an impact. *CRD*, which is calculated using the method given in below equation, can be used as a single statistic to estimate the performance of a caching scheme by roughly assuming that both congestion and diversity are equal.

22 $$\begin{aligned} CRD= \sum _{k=1}^{M} ReqTD_{c_k} + \sum _{k=1}^{M} ResTD_{c_k} \end{aligned}$$

where $ReqTD_{c_k}$ is request message transfer delay and $ResTD_{c_k}$ is delay encountered for travelling response message for any content chunk ${c_k}$ when there are total M contents available.

#### Internal link load (ILL)

We research a statistic called link-load to look into how different policies affect network congestion. We determine a link’s load as the ratio of the total number of requests that pass through it to the total number of requests traversing the link.

Assuming, each content request and content response of size H and Q respectively, the total number of requests and responses passing through any link l are presented as $N_{H}(l)$ and $N_{Q}(l)$ respectively. The link load of link l is calculated as:

23 $$\begin{aligned} LL_l= \frac{1}{T}[|H| * N_{H}(l) + |Q| * N_{Q}(l)] \end{aligned}$$

#### Content path stretch (CPS)

The CPS is a measurement of how far a requester must travel to reach a provider, and vice versa. Specifically, the shortest path to reach from requester to respondent i.e. *SP*(*reqr*, *resp*) from all the available paths is measured as CPS. The CPS for request message $CPS_H$ and response message $CPS_Q$ is calculated as given in Eqs. (24) and  (25). The CPS utilizes the mean of $CPS_H$ and $CPS_Q$ as given in Eq. (26).

24 $$\begin{aligned}{} & {} CPS_H =\frac{1}{|C| * |SP(reqr, resp)|}\sum _{H \in C}\sum _{(a,b) \in R} |SP(a,b)| \end{aligned}$$

25 $$\begin{aligned}{} & {} CPS_Q =\frac{1}{|C| * |SP(reqr, resp)|}\sum _{Q \in C}\sum _{(a,b) \in C} |SP(a,b)| \end{aligned}$$

26 $$\begin{aligned}{} & {} CPS =\frac{CPS_H + CPS_Q}{2} \end{aligned}$$

### Simulation results

To investigate the performance of the proposed strategy, the experimental results of benchmark caching techniques such as Leave Copy Everywhere (LCE), Leave Copy Down (LCD), Cache Less for More (CL4M) and prob Cache have been examined^28,29^. In LCE strategy, the content replicas are cached by each network node on the request path. To reduce cache redundancy, LCD strategy offered caching at only node which is below the node where cache hit occurs. The CL4M strategy cache content based on node’s topological information while considering node’s betweeness centrality as a key factor for content caching. The placement of content replicas based on content caching probability was introduced by ProbCache. The requester to provider distance and the amount of free cache space along the cache path are the key likelihood factors. The random cache strategy randomly chooses cache node on cache path. The findings after simulating the aforementioned caching techniques for various performance indicators have been discussed as follows.

#### Cache hit ratio (CHR)

**Figure 2:**
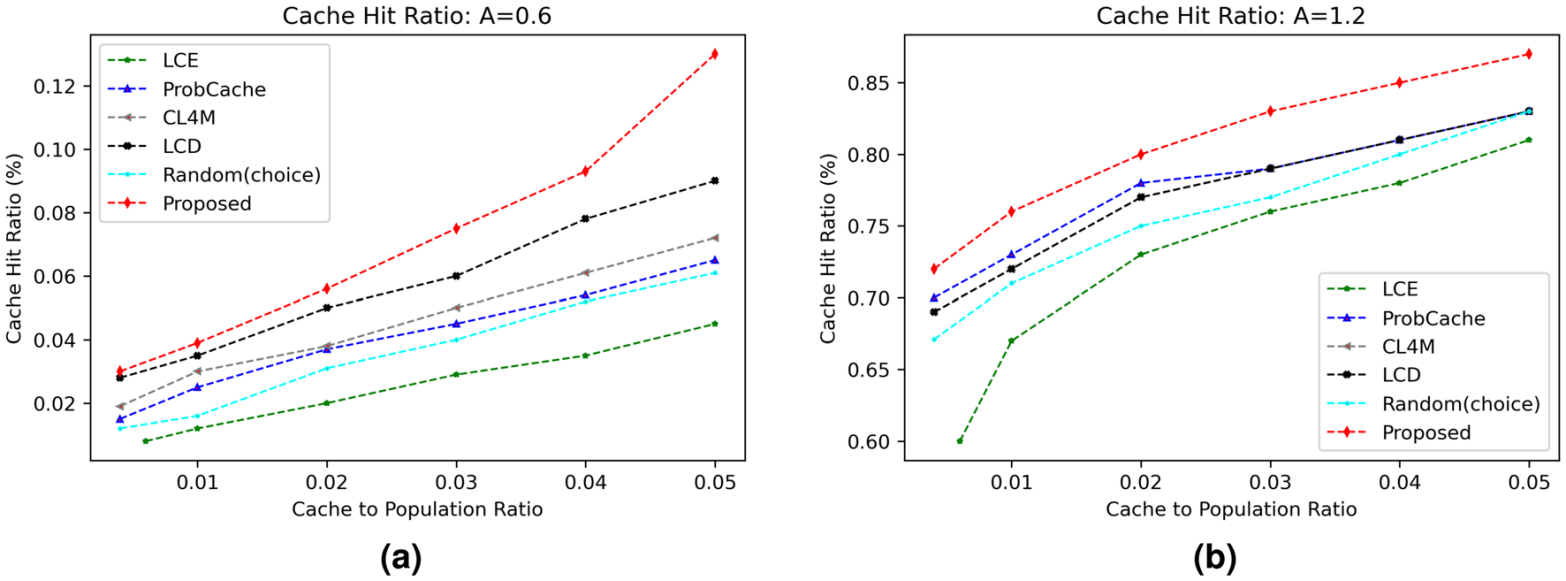
Influence of cache size on CHR.

**Figure 3:**
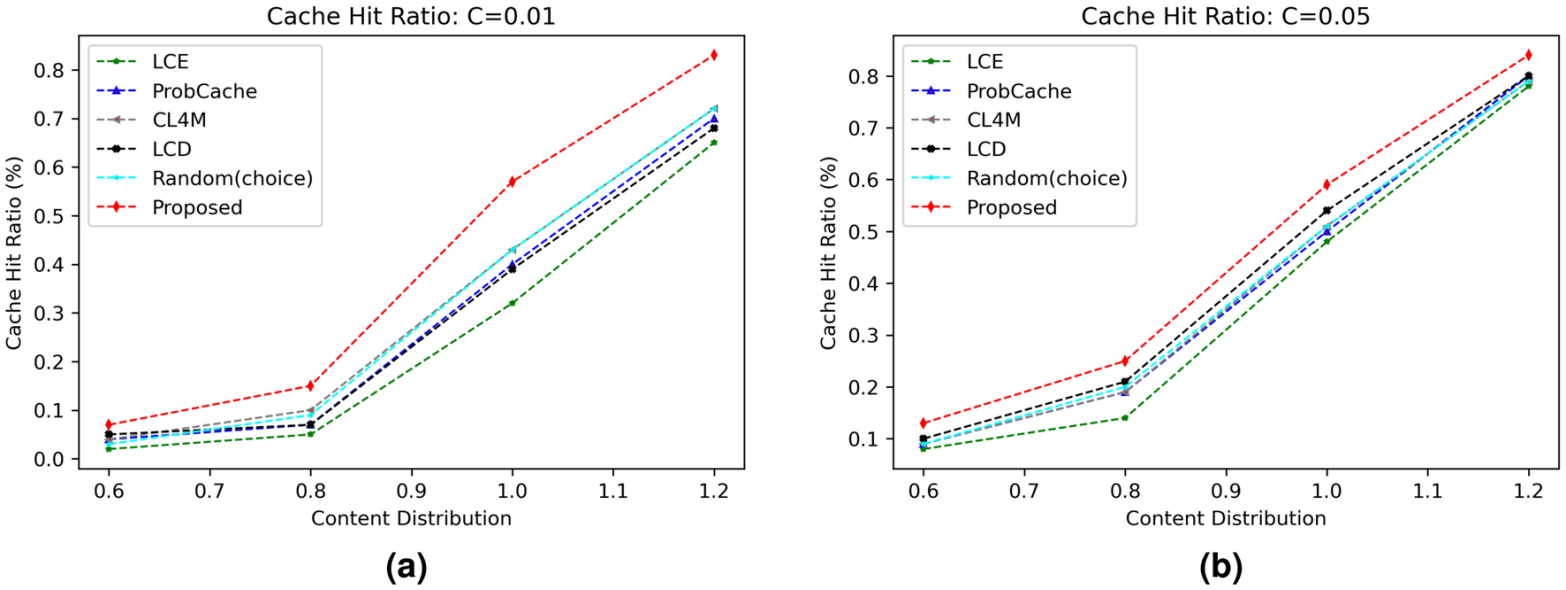
Influence of content distribution $(\alpha )$ on CHR.

The most crucial indicator for assessing the effectiveness of any caching approach is the cache hit ratio. It establishes the proportion of requests that are fulfilled out of all those that were generated. Since most requests are handled by intermediate nodes, a high CHR always implies a lighter load on the core network. The first series of experiments looks at how well the suggested CHR method performs. The outcomes of various caching algorithms for various popularity parameters $\alpha$ and cache size are shown in Figs. 2 and  3 respectively. The results show that the suggested technique performs better for greater cache hit operations than the benchmark caching solutions that are already in use due to content caching based on a probability factor. Additionally, as the content distribution skewness value increases from 0.6 to 1.2, the CHR value of the suggested system and other strategies both significantly improve (refer Fig. 2a,b). This is clear because network nodes have content caching accessible for numerous identical requests. Moreover, as more caching capacity is made available at network nodes with an increase in cache size from 1 to 5%, as shown in Fig. 3a,b, the CHR also rises.

#### Content retrieval delay (CRD)

**Figure 4:**
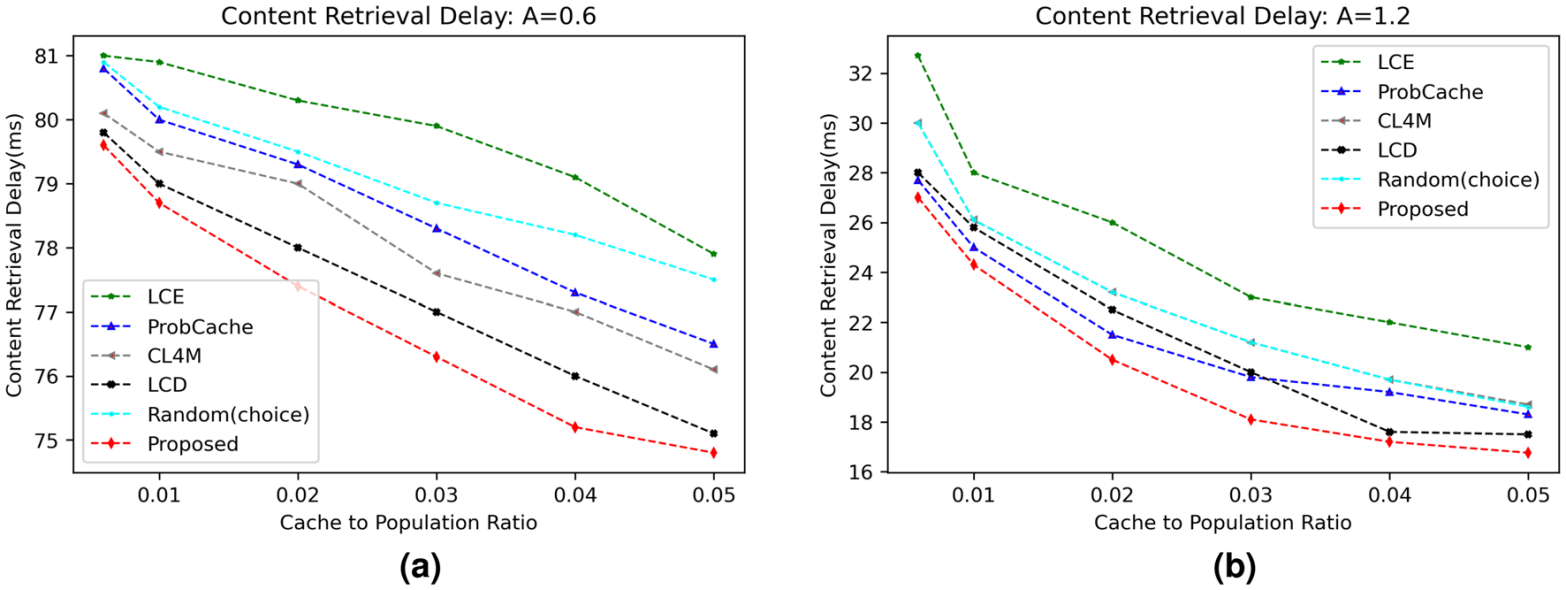
Influence of cache size on CRD.

**Figure 5:**
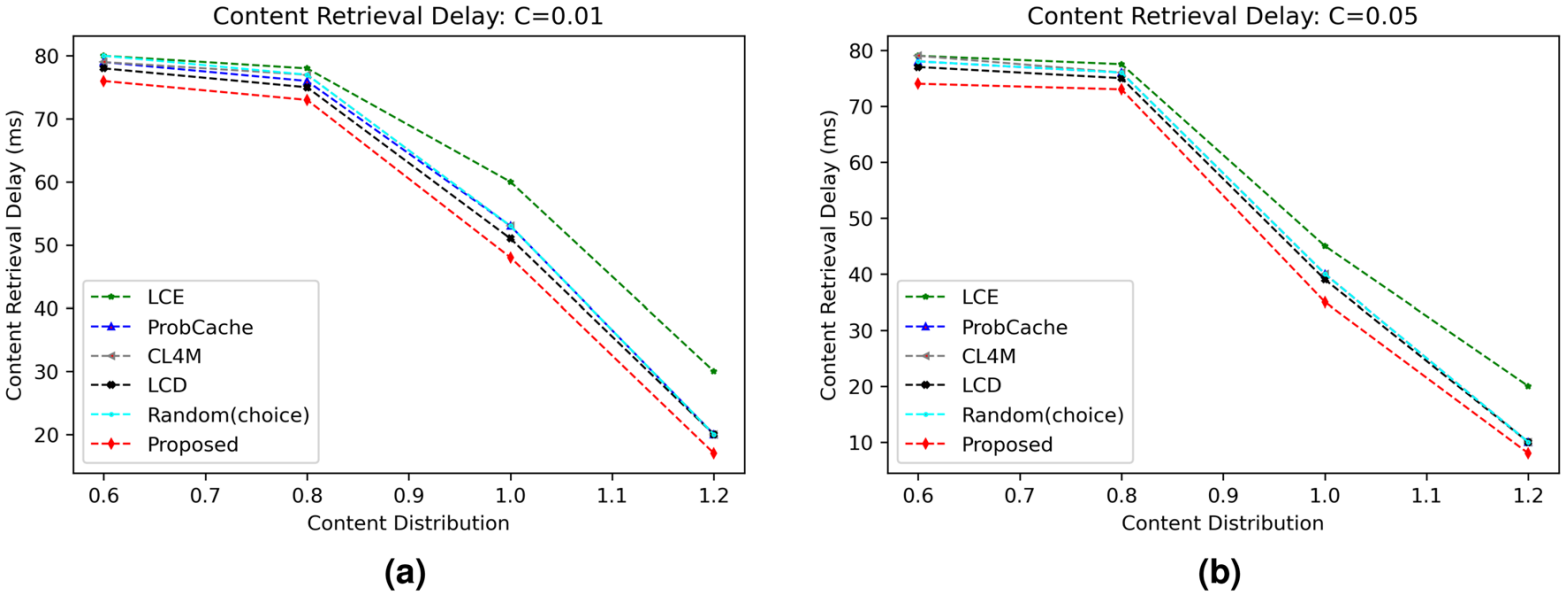
Influence of content distribution $(\alpha )$ on CRD.

The next stage of the experimentation involves analysing the data to determine how much slower the proposed strategy’s content retrieval in comparison to benchmark caching solutions. Network delay is primarily calculated using this statistic. Figures 4 and 5 exhibit the effects of various caching algorithms’ delays in content retrieval for various popularity parameters ($\alpha$) and cache sizes, respectively. The results clearly show that the suggested technique outperformed all other options for reducing retrieval delay with an increase in cache size as well as content popularity. Due to edge vehicles’ and RSUs’ increased cache capacity to store content and deliver data packets for user requests, retrieval delays are reduced as cache sizes continuously increase (refer Fig. 4a). Additionally, the data show a decreased CRD value with an increase in $\alpha$ from 0.6 to 1.2 (refer Fig. 4b). Similar to this, contents with greater probability experiences reduced latency due to caching inside network components as shown in Fig. 5a,b).

#### Content path stretch (CPS)

**Figure 6:**
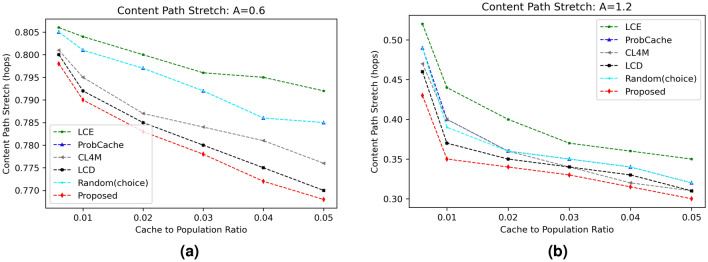
Influence of cache size on CPS.

**Figure 7:**
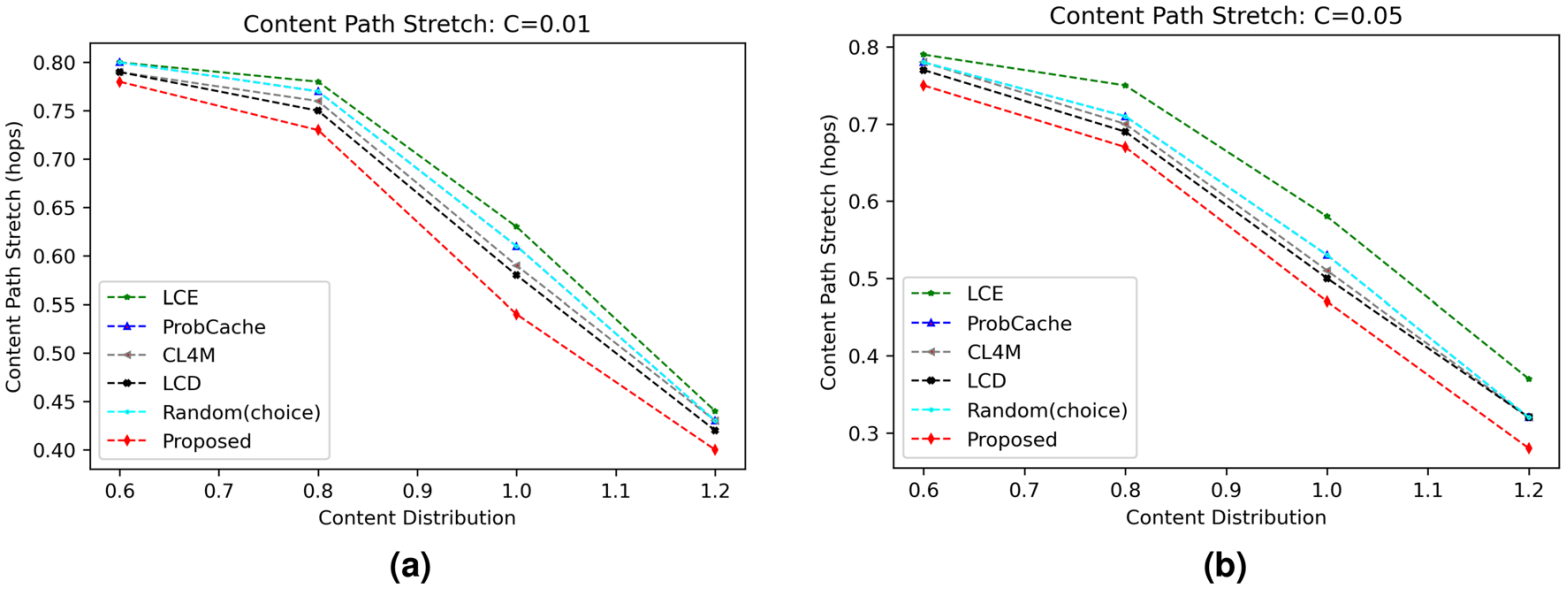
Influence of content distribution $(\alpha )$ on CPS.

The last round of experimentation shows the CPS values for various evaluated schemes under varying skewness parameter $(\alpha )$ and different cache sizes. Figures 6 and 7 represents significant examples out of total simulations with clearly indicating improved performance of the proposed scheme from rest evaluated strategies. The reduced path stretch is obviously due to caching content near to requesting vehicle using content caching probability. The results in Fig. 6a,b proved reduction in CPS for the proposed scheme as cache size increases from 1 to 5%. With increase in content distribution from 0.6 to 1.2 as shown in Fig. 6a,b, the path stretch reduces significantly due to caching available for more number of same requests. Similarly, Fig. 7a,b proved reduced CPS value of proposed scheme for cache size equal to 5% than the CPS computed at cache size equal to 1%.

#### Internal link load (ILL)

**Figure 8:**
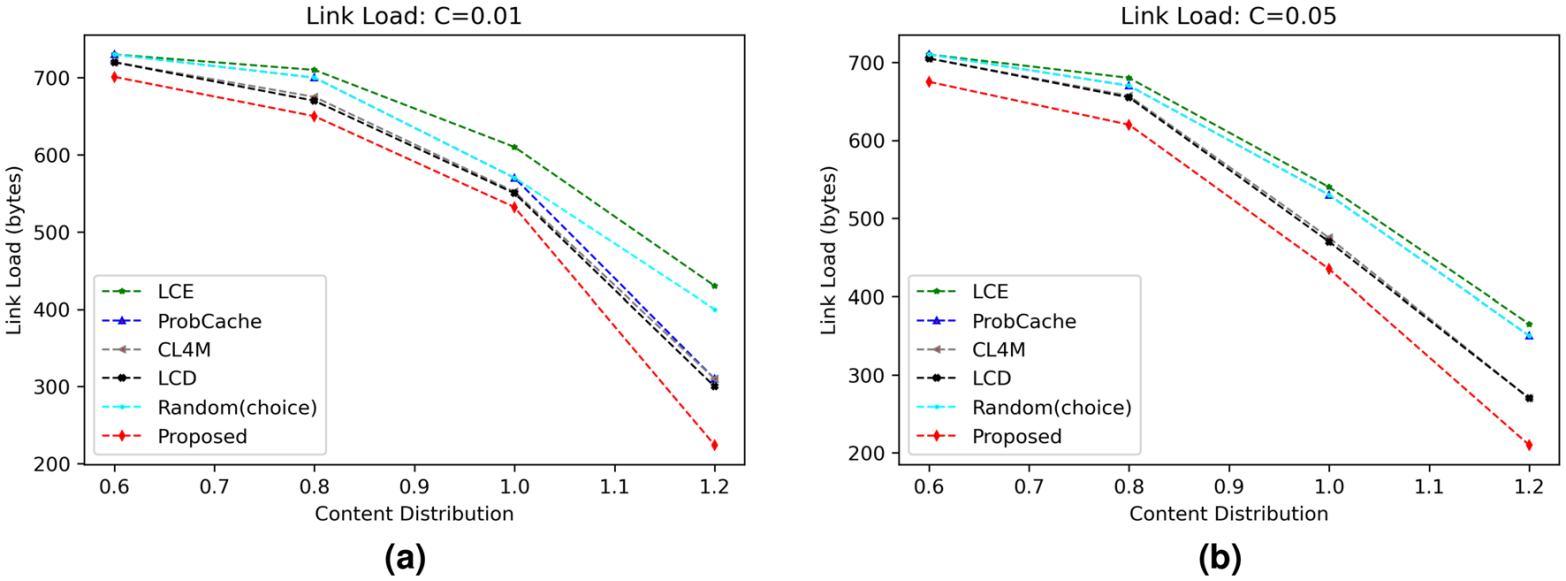
Influence of content distribution $(\alpha )$ on link load.

The link load performance of proposed strategy in contrast to various benchmark caching schemes is shown in Fig. 8. Figure 8a,b represent the link load values of different schemes with varying content distribution parameter for settled cache size of 1% and 5%, respectively. For both settings, the proposed scheme shows better results than other five compared schemes. The direct reason behind the improved performance of proposed scheme is due to caching contents based on caching probability on different nodes and therefore response can be obtained either from nearby RSU or neighboring OBU. The mean ILL of the proposed scheme for all the evaluated scenario comes to be 505.8 bytes, which is 6.76% better than the second most better scheme LCD with an average ILL of 542.5 bytes.

## Conclusion

This paper proposed a scheme for content caching at network edge to support transient content delivery in vehicular networks. To facilitate edge caching, a region based classification is performed on the vehicular network. Based on the results computed through theoretical model which considers vehicle’s velocity, region’s service area and content properties, each requesting vehicle fetch the desired content either from the nearby OBUs or from neighboring RSUs within its current region or from next region. In vehicular infotainment applications, generally requests are transient in nature^30^. The caching of transient content inside network nodes is supported by taking content caching probability into consideration. The content caching probability measures the probability of content to get cached based on content residual lifetime, its nearby availability as well as popularity. The efficiency of the proposed scheme is evaluated by performing simulations in Icarus. The results demonstrate improved performance of the proposed scheme against various benchmark caching schemes. As a future work, we will also consider price as a factor in getting content from different RSUs belonging to different network operators.

## Data Availability

All data generated or analysed during this study are included in this published article.
